# Inhibition of Rab1B Impairs Trafficking and Maturation of SARS-CoV-2 Spike Protein

**DOI:** 10.3390/v15040824

**Published:** 2023-03-24

**Authors:** Christopher Veeck, Nadine Biedenkopf, Cornelius Rohde, Stephan Becker, Sandro Halwe

**Affiliations:** 1 Institute of Virology, Philipps University Marburg, 35043 Marburg, Germany; veeckc@staff.uni-marburg.de (C.V.);; 2 German Center for Infection Research (DZIF), Partner Site Giessen-Marburg-Langen, 35043 Marburg, Germany

**Keywords:** virus–host cell interactions, Rab1B, spike protein, SARS-CoV-2

## Abstract

Severe acute respiratory syndrome coronavirus-2 (SARS-CoV-2) utilizes cellular trafficking pathways to process its structural proteins and move them to the site of assembly. Nevertheless, the exact process of assembly and subcellular trafficking of SARS-CoV-2 proteins remains largely unknown. Here, we have identified and characterized Rab1B as an important host factor for the trafficking and maturation of the spike protein (S) after synthesis at the endoplasmic reticulum (ER). Using confocal microscopy, we showed that S and Rab1B substantially colocalized in compartments of the early secretory pathway. Co-expression of dominant-negative (DN) Rab1B N121I leads to an aberrant distribution of S into perinuclear spots after ectopic expression and in SARS-CoV-2-infected cells caused by either structural rearrangement of the ERGIC or Golgi or missing interaction between Rab1B and S. Western blot analyses revealed a complete loss of the mature, cleaved S2 subunit in cell lysates and culture supernatants upon co-expression of DN Rab1B N121I. In sum, our studies indicate that Rab1B is an important regulator of trafficking and maturation of SARS-CoV-2 S, which not only improves our understanding of the coronavirus replication cycle but also may have implications for the development of antiviral strategies.

## 1. Introduction

The emergence of the severe acute respiratory syndrome coronavirus-2 (SARS-CoV-2), the causative agent of the Coronavirus Disease 2019 (COVID-19), has posed a serious threat to global public health. As of today, over 600 million cases have been reported and more than 6.5 million people died of COVID-19 [1]. Although the first specific antiviral therapies showed promising results in clinical trials [2,3,4,5,6], the potential emergence of new variants requires the identification of new antiviral targets. In order to develop novel antiviral strategies, a comprehensive understanding of the SARS-CoV-2 replication cycle is crucial.

SARS-CoV-2 particles consist of four structural proteins, including the spike protein S, the envelope protein E, the membrane protein M and the nucleoprotein N. The S protein is arranged in a homotrimeric form on the surface of the particles and mediates binding to the host cell receptor ACE2 as well as membrane fusion, which is critical for virus entry. These key features make S a primary target of humoral immune responses and the most important target for vaccines. S is a type I transmembrane glycoprotein which is proteolytically cleaved into two subunits S1 and S2, by host cell proteases such as TMPRSS2, TMPRSS11D, TMPRSS13 or furin [7,8,9,10]. Other studies have shown that S can undergo a second proteolytic cleavage by the host cell proteases cathepsin L and/or TMPRSS2 while entering the target cell [9,11]. While the N-terminal subunit S1 contains the receptor binding domain (RBD) mediating interaction with ACE2, the C-terminal subunit S2 facilitates membrane fusion. The surface of S is coated with N-linked glycans, with each trimer containing 66 potential glycosylation sites [12]. Due to its crucial role in viral replication, S has evolved to be the most thoroughly investigated protein of SARS-CoV-2. However, only little is known about intracellular trafficking and the role of host factors involved in the transport of S.

The transmembrane protein S is synthesized at the rough endoplasmic reticulum (rER). During synthesis, high-mannose oligosaccharides are covalently linked to conserved N-glycosylation sites NxS/T [13]. Subsequently, the glycoprotein concentrates close to COPII proteins near the ER exit site (ERES) and is transported from the ER to the Golgi complex crossing the ER-Golgi intermediate complex (ERGIC), a transient cargo container between the ER and Golgi complex [14]. Once S is in the Golgi complex, mannose residues are trimmed and N-Acetylglucosamine (GlcNAc), galactose and sialic acids are added to form complex-type glycans in the median Golgi complex [15]. In the trans- Golgi network, the S protein is cleaved by furin and furin-like proteases at the S1/S2 cleavage site transforming the protein from its full-length form, S0, into S1 and S2 subunits [7,8].

Forward transport of organized cargo from the ERES is mediated by the GTPases Rab1A and Rab1B as well as COPII, Sec13/31 and Sec23/24 [16,17,18]. Previous studies have shown that Rab1A participates in bidirectional movement between the ER, ERGIC and the Golgi [19], while Rab1B appears to be more stationary at the ERGIC and is frequently associated with the ERES and cis-Golgi markers such as GM130 [20]. Both Rab-GTPases are important for Golgi organization, and thus, disturbance of the Rab1 system impairs the integrity of the Golgi apparatus [21]. However, the role of Rab1A or Rab1B in the intracellular trafficking of coronavirus proteins that cross the ER/Golgi system remains unclear. In infected cells, the interaction of S, M and E leads to the retention of the structural proteins in the ERGIC, where budding and particle assembly occurs [22]. While retrograde transport and retention in the ERGIC have been extensively investigated [23,24,25,26,27], the detailed process of forward transport of newly synthesized S is largely unknown.

In this study, we aimed to characterize the early transport steps of the newly synthesized SARS-CoV-2 S from the ER to the Golgi. The focus of our interest was the role of Rab1 GTPases. We hypothesized that Rab1A and Rab1B play a central role in the trafficking and, consequently, the maturation of S. Indeed, we found that Rab1A and Rab1B colocalize with S in transfected as well as SARS-CoV-2-infected cells. Overexpression of dominant-negative (DN) Rab1B-N121I [28] led to a perinuclear accumulation of S and diminished its plasma membrane localization, while overexpression of DN Rab1A-S25N [29] had almost no effect on the distribution of S. Furthermore, intracellular levels of S were significantly downregulated upon co-expression of DN Rab1B-N121I pointing to a perturbed maturation and degradation of the protein. In addition, both DN Rab1B-N121I and DN Rab1A-S25N reduced the amount of released S. In summary, our findings indicated an important role of Rab1B in intracellular trafficking, maturation and release of the S protein of SARS-CoV-2.

## 2. Materials and Methods

### 2.1. Cell Culture and Reagents

HEK293 stably expressing ACE2 (HEK293^ACE2^, kindly provided by Shinji Makino, UTMB, Galveston, TX, USA) and HuH7 cells (matching the STR reference profile of HuH-7) were cultured in Dulbecco’s modified Eagle’s medium (DMEM, Thermo Fisher Scientific, Waltham, MA, USA) supplemented with penicillin (50 U/mL), streptomycin (50 μg/mL) (P/S) and 2 mM glutamine (Q) (abbreviated as DMEM++) and 3 or 10% fetal calf serum (FCS). HuH7 cell line was authenticated by DNA profiling of eight highly polymorphic regions of short tandem repeats in 2016 by the “Leibniz-Institut DSMZ (Deutsche Sammlung von Mikroorganismen und Zellkulturen) GmbH” (Braunschweig, Germany).

### 2.2. Antibodies

All antibodies were diluted in PBS with 1% skimmed milk. Human monoclonal antibodies against SARS-CoV-2 S (S2 subunit) were kindly provided by Florian Klein, University of Cologne (Köln, Germany) (HbnC5t1p1_B10, HbnC5t1p1_E5). We stained cis-Golgi compartments with a monoclonal mouse anti-GM130 antibody (BD Bioscience, #610822, dilution 1:5) and ERGIC with a monoclonal mouse anti-LMAN1 antibody (Invitrogen, #PA1-074, dilution 1:50). Myc-tagged Rab1A and Rab1B proteins were stained with a rabbit anti-Myc antibody (Cell Signaling, #2272S, dilution 1:50 for immunofluorescence assays (IFAs) and 1:250 for Western blot). Endogenous tubulin was stained with a monoclonal antibody from Sigma-Aldrich (#T9026, dilution 1:500). Alexa Fluor-labeled secondary antibodies were purchased from Invitrogen (anti-human-Alexa Fluor 488, #A-11013; anti-mouse-Alexa Fluor 568, #A-11004; anti-mouse-Alexa Fluor 594, #A-11032; anti-rabbit-Alexa Fluor 594, #A-11012; anti-rabbit-Alexa Fluor 647, #A27040, secondary antibodies were diluted 1:400). For Western blot analyses, following HRP-coupled secondary antibodies were used: Anti-human (Invitrogen, Waltham, MA, USA, #PA1-28587, dilution 1:3000), anti-rabbit (Dianova, Geneva, Switzerland, #711-036-152, dilution 1:40,000) and anti-mouse (Dako, Glostrup, Denmark, #P044701-2 dilution 1:40,000).

### 2.3. Plasmids

Codon-optimized (*homo sapiens*) cDNA encoding the SARS-CoV-2 S of isolate Wuhan-Hu-1 (GenBank accession number MN908947) was synthesized at Eurofins and subcloned into the pCAGGS expression plasmid using XhoI and NheI restriction sites (pCAGGS-S). A cDNA encoding C-terminally myc-tagged SARS-CoV-2 S (pCAGGS-S-myc) has been described before [7]. The plasmids pCMV-Rab1A-myc, pCMV-Rab1A-S25N-myc, pCMV-Rab1B-myc and pCMV-Rab1B-N121I-myc were a kind gift from Reinhild Prange, University of Mainz, Mainz, Germany.

### 2.4. Transfection of Plasmids in HuH7 and HEK293^ACE2^ Cells

For transient expression of SARS-CoV-2 S, 60–80% confluent HuH7 or HEK293^ACE2^ cells were co-transfected with pCAGGS-S and either pCMV-Rab1A-myc, -Rab1B-myc, -Rab1A, -S25N-myc, -Rab1B-N121I-myc or pCAGGS using TransIT transfection reagent (Mirus Bio, Madison, WI, USA, MIR 2300) according to manufacturer’s protocol. At four hours post-transfection, the culture medium was changed to DMEM++/3% fetal calf serum (FCS). At 24 h post-transfection, cells were washed with PBS and subjected to SDS–PAGE and Western blot analysis or prepared for immunofluorescence staining as described below.

### 2.5. SDS–PAGE and Western Blot Analysis

At 24 h post-transfection, cells were harvested and centrifuged for 5 min at 8000× *g*. Subsequently, cells were washed with PBS, lysed in 50 µL TGH lysis buffer (50 mM HEPES pH 7.4, 10% glycerol, 1% Triton X-100, 4% Complete and 1% PMSF in PBS) for 20 min and centrifuged again for 10 min at 14,000 rpm. Then, 40 µL of supernatant was supplemented with reducing SDS–PAGE sample buffer and heated at 95 °C for 10 min. Proteins were then subjected to SDS–PAGE (10% poly acrylamide gel) and transferred to a 0.2 µm polyvinylidene difluoride (PVDF) membrane (GE Healthcare, Chicago, IL, USA). Detection was performed using primary antibodies and species-specific HRP-coupled secondary antibodies. Proteins were visualized using the ChemiDoc XRS system and protein bands were quantified by Image Lab software (Bio-Rad, Hercules, CA, USA).

### 2.6. Immunofluorescence (IF) and Confocal Laser Scanning Microscopy (CLSM)

At 24 h post-transfection, cells were fixed for 30 min with 4% PFA/DMEM, permeabilized with 0.1% Triton X-100 for 10 min and stored at 4 °C in blocking buffer (2% BSA, 5% Glycerin, 0.1% Tween20 and 0.05% NaN_3_ in PBS). Cells were incubated for 1 h with the corresponding antibodies (diluted in blocking buffer), washed three times with PBS, and stained for 1 h with fluorescently labeled secondary antibodies and DAPI. Cells were then washed three times with PBS and mounted in FluorSave^TM^ reagent (Sigma-Aldrich, St. Louis, MO, USA). Image acquisition was performed using a confocal laser scanning microscope (CLSM) Leica TCS SP5 II and a 63x objective. Acquired pictures were analyzed and processed with ImageJ/Fiji v.1.52i.

### 2.7. Colocalization Analysis (Pearson Correlation Coefficient, PCC)

Original CLSM-obtained, unprocessed pictures were analyzed for overlapping signals with ImageJ/Fiji v.1.52i. Unprocessed layers of red and green channels were compared with a pixel-based analysis and summarized in a Pearson’s correlation coefficient (PCC) using the Coloc2 tool with Coste’s threshold regression. PCCs were collected in a data frame and statistically analyzed in RStudio v.2022.07.0 Build 548. Data were analyzed for normal distribution and variance. Due to insufficient homogeneity, *p*-values were obtained via a pairwise Wilcoxon test.

### 2.8. Deglycosylation Assay

Endogylcosidase H (Endo H, NEB) and Peptide:N-glycosidase F (PNGase F, NEB) treatment was performed according to the manufacturer’s recommendations. Briefly, lysates of transfected cells were mixed with denaturing glycoprotein buffer and heated at 100 °C for 5 min. Subsequently, 20 units of PNGase F or Endo H were added to samples in a final volume of 20 μL with NP-40 and buffer. The reaction mixtures were incubated for 1 h at 37 °C, before samples were used for Western blot analysis.

### 2.9. Ultracentrifugation

At 72 h post-transfection, cell culture supernatants were harvested and centrifuged at 10,000 rpm for 10 min to remove cell debris. Subsequently, 1.8 mL of supernatant was transferred to an SW60 ultracentrifuge tube and underlaid with 500 µL 20% sucrose. The tubes were carefully filled up with TNE buffer (50 mM Tris–HCl pH 7.4, 100 mM NaCl, 0.1 mM EDTA) and centrifuged in an SW60 rotor for 5 h at 40,000× *g* in an Optima™ L-80 XP ultracentrifuge (Beckman, Brea, CA, USA). Pellets were resuspended in 50 µL SDS sample buffer by pipetting up and down for at least 30 times.

### 2.10. Transfection of SARS-CoV-2 Infected Cells

All work with infectious SARS-CoV-2 was conducted at the biosafety level 4 (BSL4) laboratory at the Philipps University Marburg (Marburg, Germany). HEK293^ACE2^ cells grown on coverslips were infected with SARS-CoV-2 (German isolate BavPat1/2020; European Virus Archive Global (EVA-G) # 026 V-03883, Grant Number 871029) at a multiplicity of infection (MOI) of 0.1 in DMEM++ for 1 h at 37 °C. Subsequently, the virus-containing inoculum was removed, and cells transfected with 1000 ng plasmid-DNA encoding pCMV-Rab1B-myc, -Rab1B-N121I-myc or pCAGGS using TransIT transfection reagent (Mirus Bio) according to manufacturer’s protocol. After 4 h, cells were incubated with DMEM++/3% FCS at 37 °C and 5% CO_2_ in a humidified atmosphere. At 24 h post-transfection (hpt), cells were fixed twice with 4% paraformaldehyde (PFA) for 24 h before IF staining.

## 3. Results

### 3.1. Subcellular Localization of SARS-CoV-2 Spike Protein

#### 3.1.1. Intracellular Distribution of the Spike Protein

**Figure 1:**
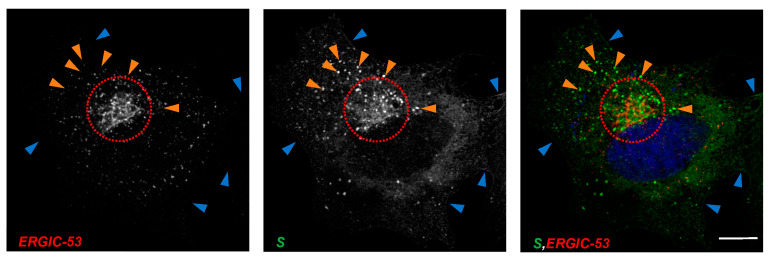
SARS-CoV-2 S colocalizes with ERGIC-53 in HuH7 cells. Subconfluent HuH7 cells were transfected with 500 ng pCAGGS-S and fixed after 24 h. Scale bars indicate 10 µm. Subcellular distribution of the S protein in HuH7 cells was stained for S protein (green), endogenous ERGIC-53 (red) and nuclei (blue) with DAPI. (i) Encircled areas mark perinuclear accumulation, (ii) orange arrows demonstrate vesicle-like structures and (iii) blue arrows mark localization at the plasma membrane. Encircled area highlights the region of colocalization. Single staining of S can be found in Figure S4A. Data are representative of three independent experiments.

To analyze the distribution of S, we recombinantly expressed SARS-CoV-2 S in HuH7 cells and characterized its subcellular localization using CLSM. As shown in Figure 1, S showed a heterogeneous distribution pattern, mainly at three sites: (i) in the perinuclear space resembling the ER-ERGIC-Golgi system (Figure 1, inside the dashed circle), (ii) vesicle-like structures throughout the cytoplasm (Figure 1, orange arrows) and (iii) delineating the plasma membrane (Figure 1, blue arrows), which is in line with published data by other groups [14,30]. Since previous studies showed evidence that coronavirus assembly takes place at the ERGIC, we further characterized the intracellular localization of SARS-CoV-2 S by simultaneously staining S and endogenous ERGIC-53, an ERGIC marker protein [31]. We found that S partially colocalized with ERGIC-53 in the perinuclear region (Figure 1, inside the dashed circle), pointing to an at least transient localization in the ERGIC, most likely caused by the C-terminal retention signal KxHxx in S [23,25].

#### 3.1.2. Maturation of the Spike Protein

**Figure 2:**
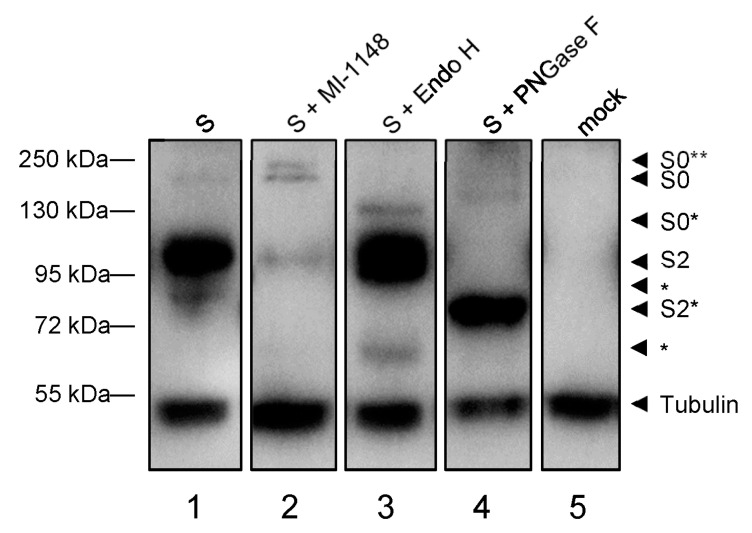
Biochemical analysis of SARS-CoV-2 S maturation via western blot analysis. Subconfluent HuH7 cells were transfected with 2 µg C-terminally myc-tagged pCAGGS-S and incubated in the absence (lanes 1, 3, 4) or presence of the furin inhibitor MI-1148 (lane 2). Cells were harvested after 24 h and lysates treated with either Endo H (lane 3) or PNGase F (lane 4) for 1 h. Immunoblots were stained for S (anti-S2) and tubulin. *: S2 subunits of unkown origin. S0*: degycosylated S0. S0**: uncleaved S0 with complex glycosylation. S2*: deglycosylated S2. Data are representative of two independent experiments.

During its transport through ER, Golgi and trans-Golgi network (TGN) S is co-translationally modified by N-glycosylation and trimming of the attached N-glycans and cleaved into the subunits S1 and S2 by the proprotein convertase furin. Thus, the characteristics of the S-associated N-glycans and the appearance of S cleavage products can be used to determine the localization of S in ER, Golgi or TGN. To analyze the maturation of S, myc-tagged S was ectopically expressed in HuH7 cells in the presence or absence of the furin inhibitor MI-1183 [32] and cells were lyzed at 24 hpt. Cell lysates were treated with endoglycosidase H (Endo H), Peptide:N-glycosidase F (PNGase F) or left untreated and analyzed via Western blot using an S2-specific human monoclonal antibody (Figure 2).

As depicted in Figure 2, lane 1, the S2-specific human monoclonal antibody detected two prominent bands. A dominant form of approximately 95 kDa, which was resistant to Endo H and changed its migration pattern only upon treatment with PNGase F (Figure 2, lanes 1–3, S2*). This band is suggested to represent S2, which had been transported across the median Golgi and thus gained Endo H resistance (Figure 2). The second band recognized by the S2-specific antibody migrated at 180 kDa and was sensitive to treatment with Endo H, which resulted in a faint band migrating at approx. 130 kD (Figure 2, S0*). Endo H-sensitivity indicated that this S-specific protein had not passed the median Golgi, leading to the conclusion that this 180 kD protein represented the immature and uncleaved form S0 [33]. When the cells expressing S had been incubated with MI-1148, an inhibitor of the protease furin, the signal intensity of S2 was dramatically weakened while an increase of S0 intensity was recorded, suggesting that the cleavage of S0 was, indeed, inhibited. Another form of S emerged due to MI-1148 treatment (lane 2, S0**), which might represent uncleaved S0 with complex-type N-glycans. It is possible that this form is visible only in the presence of MI-1148 because it is otherwise immediately cleaved into S1 and S2. More bands smaller than 95 kDa (*) of unknown origin that have not been further characterized can be detected and might differ between different cell lines (Supplementary Figure S1). Due to these differences in cell lines, all following experiments were conducted with HuH7 cells for coherency purposes, except for the infection studies.

### 3.2. Rab1B Is Critical for Transport, Maturation and Release of SARS-CoV-2 S

#### 3.2.1. SARS-CoV-2 Spike Protein Colocalizes with Rab1A and Rab1B

Rab-GTPases are essential regulators of specific intracellular transport pathways. More than 60 Rab-GTPases have been described so far, which are localized specifically at different cellular membrane compartments guiding the transport of vesicles and can therefore be used as markers for trafficking pathways and subcellular compartments [34,35]. The two isoforms Rab1A and Rab1B are described to be master regulators of the ER/ERGIC/Golgi interface [21]. Thus, we hypothesized that either Rab1A, Rab1B or both are involved in ER-to-Golgi trafficking of S. In this study, we used DN mutants of Rab1A and Rab1B, which have an increased affinity for GDP and are, therefore, in a constant inactive state [28,29].

**Figure 3:**
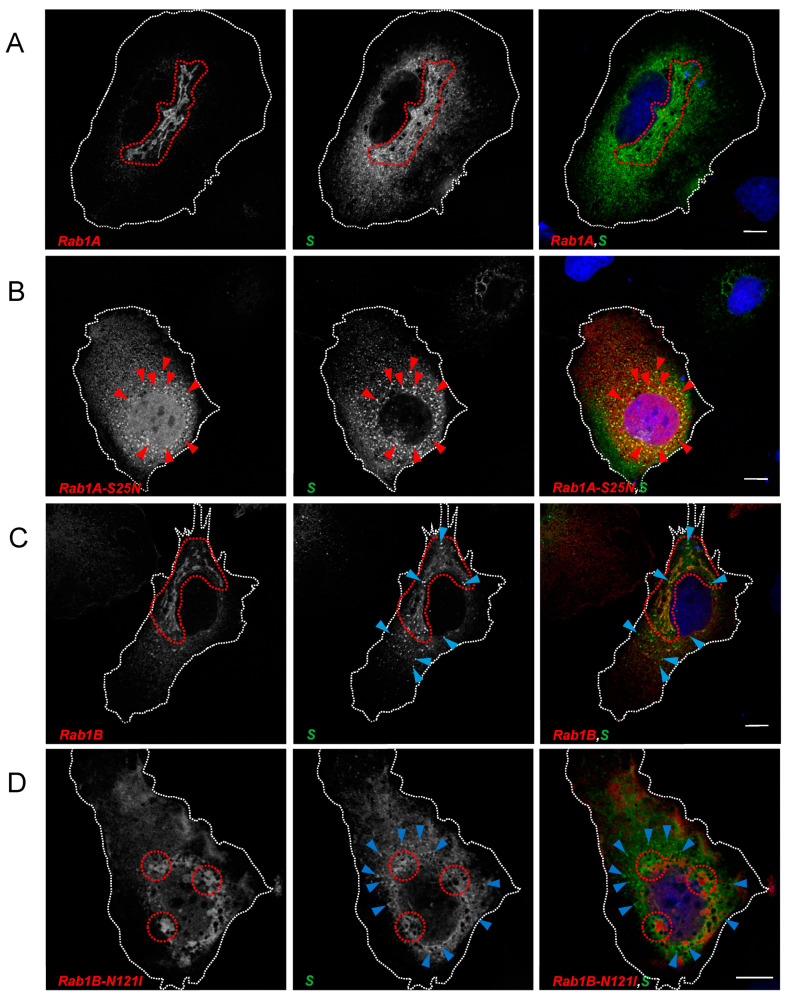
Rab1A and Rab1B are involved in SARS-CoV-2 S transport. Subconfluent HuH7 cells were transfected with 500 ng pCAGGS-S and 1000 ng (**A**) pCMV-Rab1A-myc, (**B**) pCMV-Rab1A-S25N-myc, (**C**) pCMV-Rab1B-myc or (**D**) pCMV-Rab1B-N121I-myc, respectively. Cells were fixed 24 hpt and stained for SARS-CoV-2 S (green), Rab1A/Rab1B (anti-myc, red) and nuclei (blue) with DAPI. Scale bars indicate 10 µm. Encircled areas in (**A**–**C**) highlight regions of colocalization between S and Rab1 variants. Red arrows in (**B**) point to distinct vesicular structures where S and Rab1A-S25N colocalize. Blue arrows in (**C**) point at vesicle-like structures of S that do not colocalize with Rab1B. In (**D**), encircled areas highlight the close proximity of S and DN Rab1B-N121I. Blue arrows point to accumulations of punctate structures that do not colocalize with Rab1B-N121I. Data are representative of three independent experiments.

HuH7 cells were transfected with plasmids encoding S and different myc-tagged forms of Rab1A and Rab1B. After fixation at 24 hpt and staining with anti-S2 and anti-myc antibodies, we could observe strong colocalization of S and Rab1A in perinuclear areas indicating that S traversed Rab1A-positive structures in the ER/Golgi system (Figure 3A, inside highlighted area). Co-expression of Rab1A and S did not change the distribution of either protein. In contrast, co-expression of DN Rab1A-S25N and S led to a redistribution of S with apparently increased colocalization of the two proteins in peripheral punctate structures (Figure 3B, red arrows) and a slight reduction of S in the cellular cortex and at the plasma membrane.

Compared to Rab1A, the colocalization of S and Rab1B was less pronounced. Probably, most of the overexpressed Rab1B is concentrated in the ER. Therefore, colocalization with S might be more transient due to rapid transport to the ERGIC and the Golgi. Nonetheless, overlapping regions between Rab1B and S close to the nucleus (Figure 3C, inside highlighted area) and a seeming increase of S-positive vesicle-like structures compared to S without Rab1B-overexpression could be observed (Figure 3C, blue arrows). The latter might be explained by increased trafficking of S due to the overexpression of Rab1B. Intriguingly, DN Rab1B-N121I had a strong impact on the distribution of the S protein. As shown in Figure 3D, following overexpression of DN Rab1B-N121I, S (i) aggregated in close proximity to DN Rab1B-N121I (encircled areas) without being colocalized and (ii) accumulated in scattered peripheral compartments (blue arrows) without colocalization with Rab1B-N121I. S was hardly detectable at the plasma membrane, indicating an abrogated transport of S in the early secretory pathway when Rab1B function was perturbed.

To confirm our observations, we performed a quantitative analysis of overlapping fluorescence signals of Rab1 and S. For this purpose, we obtained Pearson’s correlation coefficients (PCC)—a pixel-based quantification index comparing the correlation of overlapping pixel intensities from two channels. This index is summed up in a non-dimensional value between −1 (no correlation), 0 (arbitrary correlation) and 1 (total correlation).

**Figure 4:**
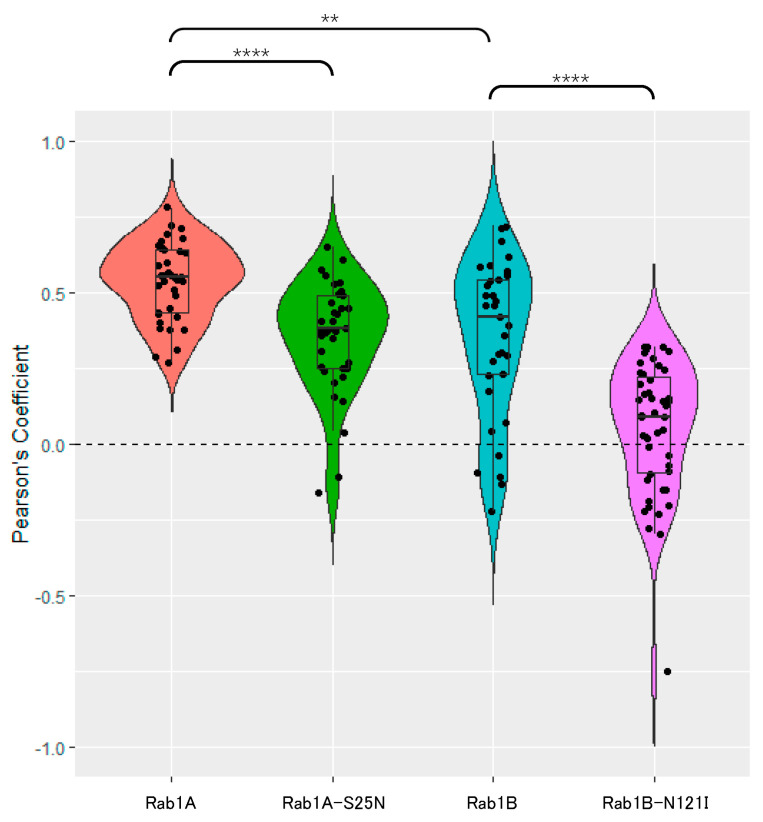
Quantification of Rab1 and S colocalization confirms observations from IFAs. For evaluation of the PCC, 30 to 50 cells from at least four independent experiments were analyzed, and colocalization of red and green signals in a defined region of interest was quantified with Coste’s threshold regression. Obtained data are visualized as a boxplot. Statistical significance was obtained via a pairwise Wilcoxon test due to insufficient homogeneity. **: *p* < 0.01, ****: *p* < 0.0001.

As depicted in Figure 4, Rab1A shared the most overlapping signals with S (mean PCC = 0.539). While the distribution of S after co-expression of Rab1A-S25N is affected, signals still overlap in a considerable amount of areas (mean PCC = 0.355). As expected, Rab1B also shares substantial colocalization with S, comparable to Rab1A and Rab1A-S25N (mean PCC = 0.348). Interestingly Rab1B-N121I behaves very differently regarding colocalization with S. The mean PCC dropped to 0.051, while the effect of Rab1B-N121I on co-expressed S was strongest. We observed an accumulation of S in close proximity to Rab1B-N121I in perinuclear areas but no direct colocalization. While in the presence of Rab1B-N121I the number of S-positive vesicle-like punctate structures increased, no colocalization between the two proteins could be observed. The drastic changes in the distribution of S after co-expression with Rab1B-N121I point to an important role of Rab1B for S trafficking pathways.

#### 3.2.2. Functional Rab1B Is Necessary for SARS-CoV-2 Spike Protein Maturation

As a glycoprotein, trafficking stages of S can be determined by the properties of the more than 20 N-glycosidic oligosaccharides, which are co-translationally attached to the protein. Initially, mannose-rich oligosaccharides are added to the protein in the ER, which can be removed in vitro by treatment with endoglycosidase (Endo) H leading to a reduced molecular mass. When S passes the medial Golgi cisterna, Endo H-sensitive oligosaccharides are modified by a complex system of glycosidases and glycosyltransferases, leading to the formation of complex-type oligosaccharides, which thus gain resistance to Endo H but are sensitive to endoglycosidase PNGase F. In the trans-Golgi-network, S is cleaved into the subunits S1 and S2 by the proprotein convertase furin [8]. These transport-dependent and sequentially occurring modifications of S were then used to further narrow down at which step DN Rab1B-N121I impaired the transport of S. We hypothesized that the blockage of S trafficking prior to the Golgi apparatus should result in an altered maturation and cleavage of S.

To test this hypothesis, we co-expressed S with DN mutants of Rab1A and Rab1B, harvested the cells and digested the lysates with Endo H or PNGase F; the latter removed all forms of N-linked glycans and served as a control for the overall presence of N-linked glycosylation.

**Figure 5:**
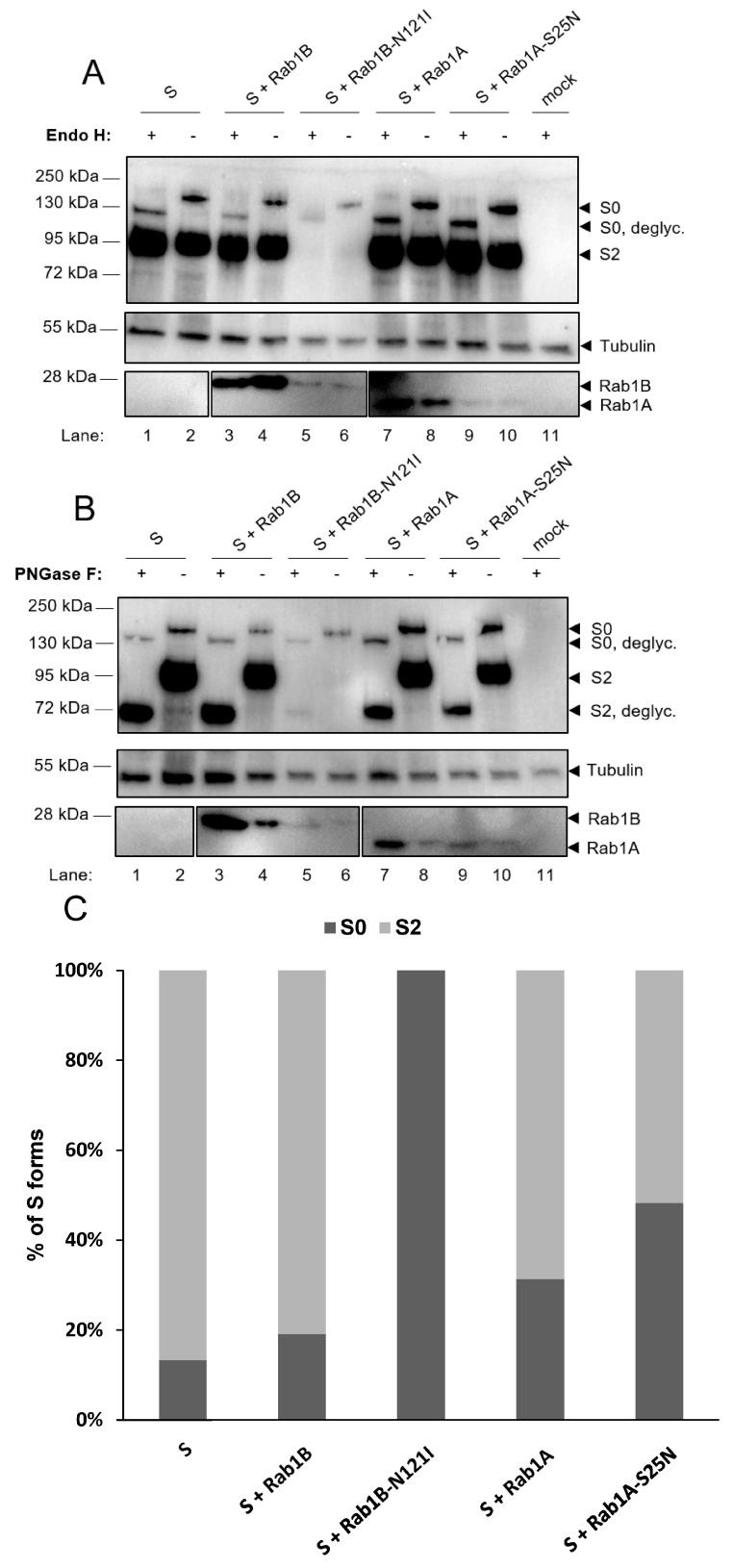
Rab1B-N121I efficiently inhibits maturation of SARS-CoV-2 S. Subconfluent HuH7 cells were transfected with 1 μg pCAGGS-S and 3 μg pCMV-Rab1A-myc and pCMV-Rab1B-myc; the corresponding DN mutants or empty vector pCAGGS as a control, respectively. Cells were harvested at 24 hpt and cell lysates were treated with either (**A**) Endo H or (**B**) PNGase F for 1 h. Immunoblots were stained for S, Rab1A/Rab1B (anti-myc) and tubulin. (**C**) Levels of S0, S2 and Tubulin from (**A**) were quantified and the ratio of S0 and S2 was calculated. The values in the graph represent triplicates from three independent experiments.

Digestion of single-expressed S by Endo H and PNGase F led to the same results as in Figure 2. Interestingly, co-expression of DN Rab1B-N121I and S led to an almost complete loss of S2, which indicated that inhibition of Rab1B strongly interfered with the maturation of S (Figure 5A,B, lanes 5 and 6). To quantify these observations, the ratio between S0 and S2 was calculated for each sample from Figure 5A. While S2 vanished upon co-expression with DN Rab1B-N121I, inhibition of Rab1A led to a slight though a not significant shift of S0/S2 ratio toward S0, indicating that Rab1A might also play a role in the maturation of S (Figure 5C). We observed that immunoblot signals of mutants of Rab1 were weaker than that of their corresponding wildtype Rab1. This effect might be caused by either reduced stability of the mutants or decreased expression levels. While this had obviously no implications for the effect of DN Rab1B on S, it cannot be excluded that stability problems are an explanation for the only minor effects of DN Rab1A.

In sum, our results suggested that inhibition of Rab1B prevents S from reaching the Golgi apparatus where trimming of N-linked glycans takes place and further prevents transport to the TGN, where cleavage by furin is facilitated. This effect was not observed upon co-expression of DN Rab1A-S25N and S, pointing to a specific role of Rab1B in the transport of S. We, therefore, concluded that Rab1B plays a crucial role in the maturation and ER-to-Golgi transport of S.

In line with the observed decreased amounts of S at the plasma membrane in IF analysis, inhibition of Rab1A significantly impaired the release of S by 44–59% (Supplementary Figure S2). Although this seems to be in contrast to our observation that Rab1A plays a minor role in the maturation and early trafficking of S, it is possible that Rab1A has a bigger effect on trafficking pathways that enable S to reach the plasma membrane. However, the role of Rab1A in the trafficking of S needs to be elaborated more extensively in future studies.

In sum, we observed that Rab1B played an important role in the intracellular maturation of S when ectopically expressed. Inhibition of Rab1B had a striking effect on trafficking (Figure 3) and distribution of S. This effect could be the result of a perturbed transport that inhibited the maturation and cleavage of S into subunits S1 and S2 (Figure 5).

### 3.3. Inhibition of Rab1 Disturbs the Integrity of Compartments of the Early Secretory Pathway

#### 3.3.1. Rab1 Is Important for ERGIC and Golgi Organization

**Figure 6:**
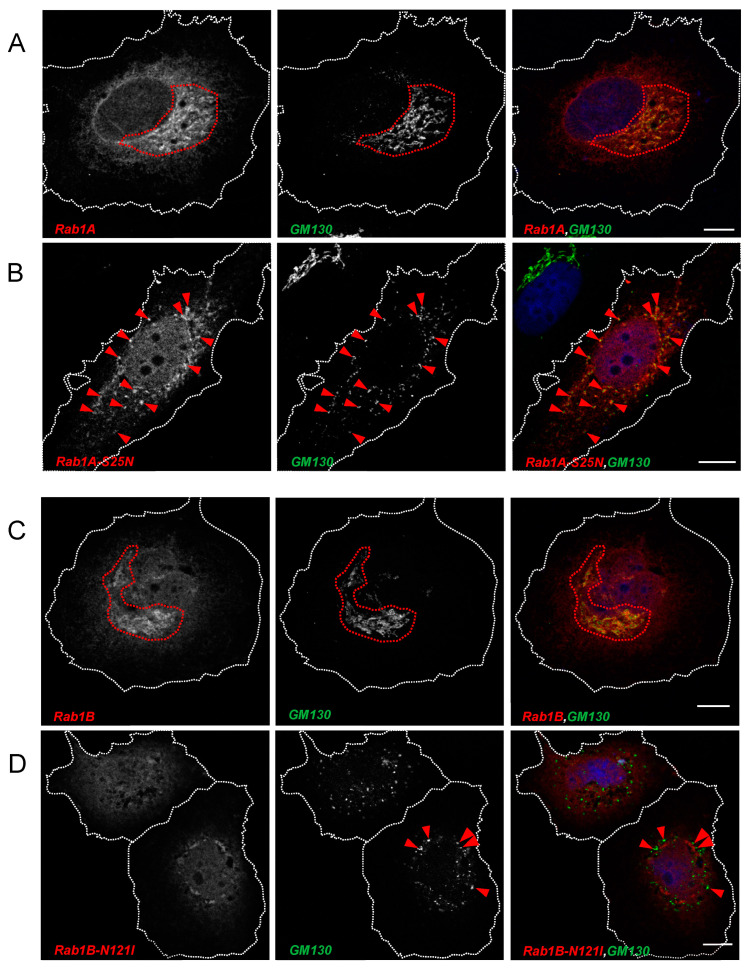
Effect of ectopic expression of Rab1A, Rab1B or the corresponding DN mutants on the distribution of endogenous GM130. Subconfluent HuH7 cells were transfected with (**A**) pCMV-Rab1A-myc, (**B**) pCMV-Rab1A-S25N-myc, (**C**) pCMV-Rab1B-myc or (**D**) pCMV-Rab1B-N121I-myc respectively. Cells were fixed 24 hpt and stained for endogenous GM130 (green), Rab1A/Rab1B (anti-myc, red) and nuclei (blue) with DAPI. Scale bars indicate 10 µm. Encircled area marks a coherent organization of the Golgi systemand arrows demonstrate a scattered Golgi system. GM130 single staining can be found in Figure S4B. Data are representative of two independent experiments.

Having demonstrated that inhibition of Rab1B perturbs transport and maturation of S, we next sought to determine whether ectopic expression of DN Rab1B has visible consequences on the distribution of marker proteins of the early secretory pathway. Thus, we further characterized the localization of Rab1A and Rab1B as well as the corresponding DN mutants Rab1A-S25N and Rab1B-N121I by analyzing their colocalization with GM130 and ERGIC-53. As reported by others [36], we also found that both Rab1A and Rab1B colocalize with GM130 (Figure 6A,C), which is a frequently used marker for the cis-Golgi.

After ectopic expression of either DN Rab1 mutant, we observed a change in the distribution of GM130 which seemed to indicate a reorganization of the Golgi complex (compare Figure 6A–D). While Rab1A-S25N still colocalized with GM130 (Figure 6B), DN Rab1b lost its colocalization with GM130. Interestingly, the subcellular distribution of DN Rab1A-S25N differed from Rab1B-N121I. While DN Rab1B-N121I formed very characteristic perinuclear accumulations, DN Rab1A-S25N appeared in a more scattered distribution.

**Figure 7:**
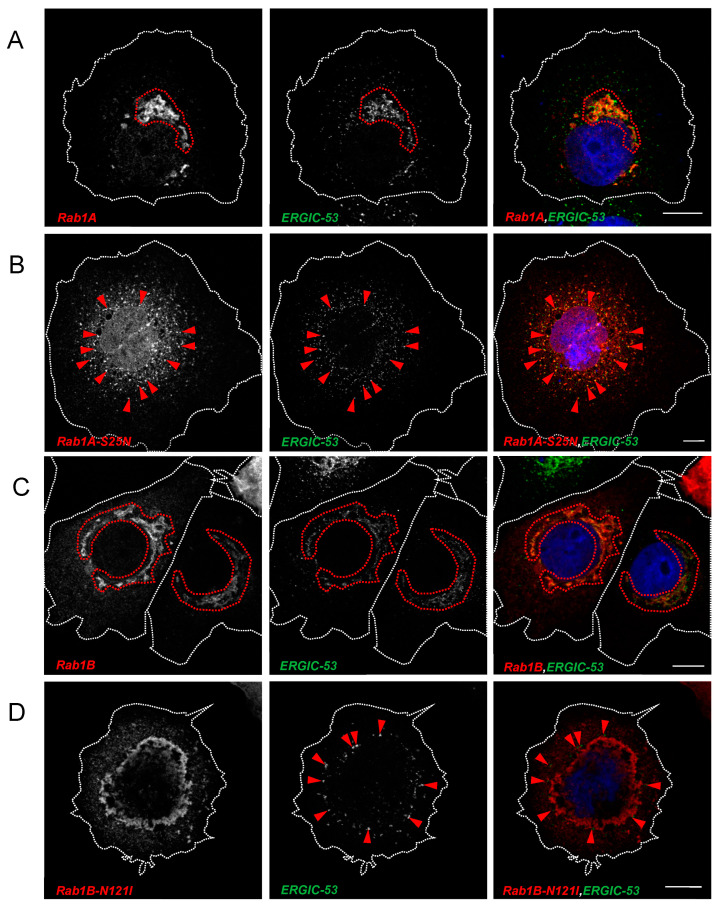
Overexpression of Rab1 DN mutants impairs the structural integrity of the ERGIC. Colocalization analysis of Rab1A, Rab1B and corresponding DN mutants in HuH7 cells with endogenous ERGIC-53. Subconfluent HuH7 cells were transfected with 750 ng (**A**) pCMV-Rab1A-myc, (**B**) pCMV-Rab1A-S25N-myc, (**C**) pCMV-Rab1B-myc or (**D**) pCMV-Rab1B-N121I-myc, respectively. Cells were fixed 24 hpt and stained for endogenous ERGIC-53 (green), Rab1A/Rab1B (anti-myc, red) and nuclei (blue) with DAPI. Scale bars indicate 10 µm. Encircled area marks organized ERGIC and arrows demonstrate scattered ERGIC. ERGIC-53 single staining can be found in Figure S4C. Data are representative of three independent experiments.

Both Rab1-GTPases colocalized with ERGIC- 53 (Figure 7A,C). Ectopic expression of DN Rab1A-S25N or DN Rab1B-N121I changed the appearance of endogenous ERGIC-53, suggesting a disruptive effect of the two DN Rab1 proteins on the ERGIC compartment. (Figure 7B,D). In the presence of DN Rab1 proteins, the distribution of ERGIC-53 was similarly changed as GM130, indicating a loss of organization upon expression of DN Rab1 mutants. The coherent and organized morphology of the ERGIC compartment turned into peripheral punctate EGIC-53-positive structures, which colocalized with DN Rab1A-S25N but not with DN Rab1B-N121I. This result suggested that while DN Rab1A-S25N is associated with the ERGIC, DN Rab1B-N121I is not (Figure 7C,E).

In sum, confocal microscopy revealed that inhibition of Rab1-dependent trafficking perturbed the integrity of both the Golgi apparatus and the ERGIC. While DN Rab1A-S25N colocalized with both GM130 and ERGIC-53, DN Rab1B-N121I did not colocalize with either of the two. Our observations are in line with published data about the effect of Rab1 DN mutants [29,37,38,39].

#### 3.3.2. Inhibition of Rab1B Traps S in the ERGIC

**Figure 8:**
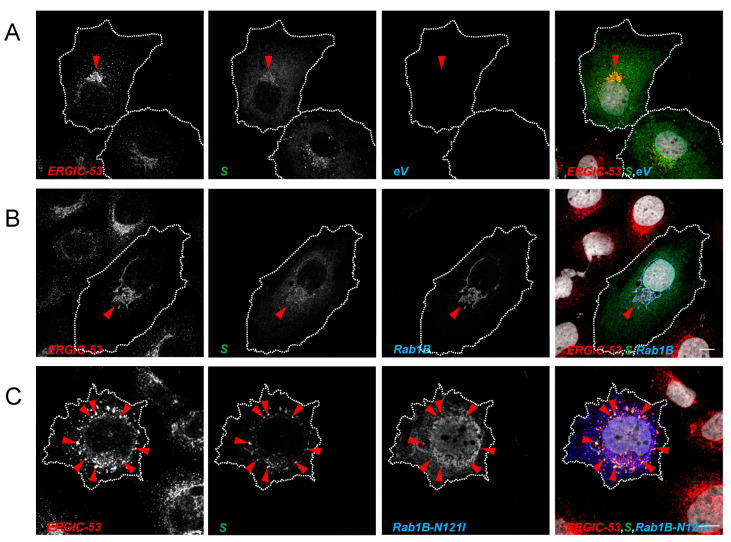
Inhibition of Rab1B traps S protein in the ERGIC. Subconfluent HuH7 cells were transfected with 500 ng pCAGGS-S and 1000 ng (**A**) empty vector (eV) pCAGGS, (**B**) pCMV-Rab1B-myc or (**C**) pCMV-Rab1B-N121I-myc, respectively. Cells were fixed 24 hpt and stained for SARS-CoV-2 S (green), ERGIC-53 (red), Rab1A/Rab1B (anti-myc, blue) and nuclei (grey) with DAPI. Scale bars indicate 10 µm. Arrows demonstrate colocalization of S and ERGIC-53. Data are representative of two independent experiments.

We were then interested in understanding at which point in the early secretory pathway the transport of S is blocked by DN Rab1B. Since expression of Rab1B-N121I blocked maturation of S in a pre-cleavage state and cleavage of S by furin takes place in the Golgi/TGN, we assumed that inhibition of Rab1B may disturb ER exit sites and therefore leads to accumulation of S in the ER. As expected, S colocalized with both Rab1B and ERGIC-53 (Figure 8B), indicating that under those conditions, S and Rab1B are both localized in the ERGIC (Figure 8B). Interestingly, when the Rab1B-dependent pathways were disturbed by the DN mutant Rab1B-N121I, ERGIC-53 and S changed their intracellular distribution but stayed colocalized. In contrast, Rab1B-N121I was not colocalized with S and ERGIC 53 (Figure 8C). This suggested that S is retained in the (modified) ERGIC structures and not in the ER (Figure 8C). DN Rab1B-N121I did not colocalize with either S or ERGIC-53. Thus, we concluded that inhibition of Rab1B leads to a loss of ERGIC integrity and traps the S in ERGIC-derived structures.

### 3.4. Rab1B Colocalizes with S in SARS-CoV-2 Infection

**Figure 9:**
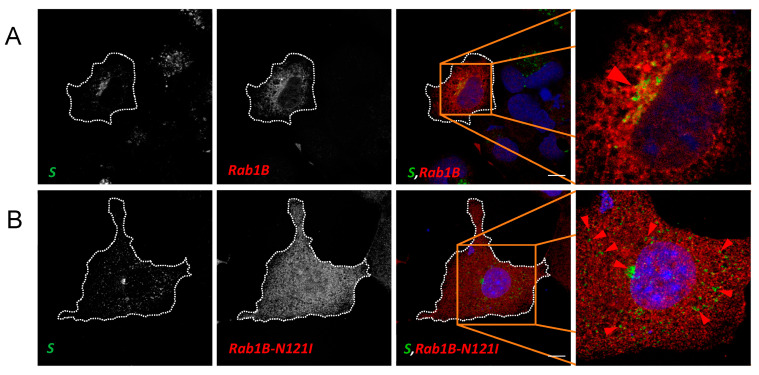
Inhibition of Rab1B leads to redistribution of SARS-CoV-2 S in infected HEK293^ACE2^ cells. Subconfluent HEK293^ACE2^ cells were infected with SARS-CoV-2 and subsequently transfected with 1000 ng (**A**) pCMV-Rab1B-myc or (**B**) pCMV-Rab1B-N121I-myc, respectively. Next, 24 hpt cells were fixed and stained for SARS-CoV-2 S (green), Rab1B (anti-myc, red) and nuclei (DAPI). Magnified region shows the difference in the distribution of S. Scale bars indicate 10 µm. Data representative of three independent experiments.

Our investigations led us to the conclusion that Rab1B is important for ERGIC-to-Golgi transport of S. Therefore, we next tested whether Rab1B shows colocalization with S in SARS-CoV-2 infected cells. To this end, we used ACE2-overexpressing HEK293 cells (HEK293ACE2) for infection experiments because HuH7 are only moderately susceptible to SARS-CoV-2 [40]. Subsequently, infected cells were transfected with Rab1B or Rab1B-N121I. At 24 hpi, cells were fixed, stained for S and Rab1B and analyzed via confocal microscopy (Figure 9).

In contrast to the ectopic expression of S (Figure 1), we observed that in infected cells, S was concentrated in a very condensed space in close proximity to the nucleus (Figure 9A and Figure S3). The difference to recombinantly expressed S might be caused by extreme retention of S due to interaction with both M and E, as described before [22]. We found more noticeable colocalization of Rab1B and S in condensed perinuclear spots (Figure 9A and Figure S3). The stronger colocalization might be caused by increased retention of S in the ERGIC as compared to recombinant expression of S alone. Interestingly, expression of DN Rab1B-N121I led to a redistribution of S and colocalization between the two proteins was completely abrogated (Figure 9B and Figure S3). We observed that S was redistributed from the perinuclear space into peripheral punctate structures.

The more diffuse distribution of Rab1B in infected cells might be explained by the lower expression level of the protein in infected compared to transfected cells since the virus employs most of the cellular protein synthesis machinery. As described above (Section 3.3.1), Rab1B is not exclusively localized in the ERGIC but rather at multiple sites of the ER-Golgi complex at the same time. The punctate distribution of S after co-expression with DN Rab1B-N121I closely resembles the observed phenotype in transfected cells. In sum, our findings in SARS-CoV-2 infected cells were in line with the results in transfected cells, indicating that Rab1B plays an important role in S trafficking in SARS-CoV-2-infected cells.

## 4. Discussion

We observed significant changes in the subcellular distribution of S after the inhibition of Rab1B and a strong inhibition of S cleavage into the two S subunits indicating a crucial role of Rab1B in the intracellular trafficking of S. In addition, in SARS-CoV-2 infected cells, we were able to show that inhibition of Rab1B led to the redistribution of S from condensed perinuclear space into scattered peripheral vesicular structures, indicating that Rab1B is involved in the trafficking of S in SARS-CoV-2 infected cells as well.

IF analysis showed colocalization of S with both Rab1A and Rab1B. However, in contrast to DN Rab1A-S25N, ectopic expression of DN Rab1B-N121I had a striking effect on the distribution of S, which accumulated in perinuclear and peripheral punctate ERGIC-53-positive structures, indicating a disruption of ERGIC integrity. It is known that in the absence of active Rab1B, ER exit sites are still formed, but the formation of a vesicular tubular system, as typical for the ERGIC, is inhibited [37]. As a consequence, the disturbed ERGIC formation leads to ER swelling, and blockage of ER-to-Golgi transport, as shown for rhodopsin in *Drosophila* [41]. Therefore, it is possible that the observed punctate ERGIC-53-positive structures represent a scattered, disintegrated ERGIC-like compartment. This would support a scenario in which S is trapped in the disintegrated ERGIC. The role of Rab1B may be less important for ER-to-ERGIC trafficking of S, but essential for ERGIC-to-Golgi trafficking. An explanation could be that Rab1B-dependent ERGIC exit sites are no longer functional, which leads to the accumulation of S in the ERGIC. Alternatively, the observed S/ERGIC-53-positive punctate structures might represent degradative compartments (e.g., lysosomes), which would also explain the low level of S2 after co-expression with DN Rab1B-N121I.

The lectin ERGIC-53 is an important cargo receptor for the trafficking of arenavirus, hantavirus, coronavirus, orthomyxovirus, and filovirus glycoproteins [42], which is in line with our findings in this work. We found subtle colocalization of recombinantly expressed S and ERGIC-53 and accumulation of both after inhibition of Rab1B-dependent pathways. This points to a potential involvement of ERGIC-53 in the trafficking of S. Further experiments are needed to allow more insights into the importance of ERGIC-53 for SARS-CoV-2 assembly and transport. For example, overexpression of DN ERGIC-53-KKAA [43] might reveal the role of ERGIC-53 for SARS-CoV-2 replication and maturation of S in future experiments.

Using an Endo H digestion assay, we further showed that in the presence of DN Rab1B, maturation of S is inhibited before entry into the Golgi system. Interestingly, despite the drastic loss of S2, we did not observe an increase of S0, as could have been expected. Immunoblot analysis revealed that S2 vanished almost completely, and levels of S0 were almost unchanged. One explanation for the level of S0 not being expanded could be that the transport blockage leads to the degradation of S0 in autophagosomal structures or in proteasomes near the ER or ERGIC-53-positive compartments [44].

It is yet unknown whether the observed effect of Rab1B on S trafficking on maturation is caused by direct interaction or an indirect mode of action. Studies suggest that the Influenza A RNP complex directly interacts with Rab11 [45,46]. However, the observed changes of the cellular transport hub in terms of rearrangement of the ERGIC and Golgi upon Rab1B pathway inhibition suggests that a loss of direct interaction between S and Rab1B is most likely not the reason for the observed effects on S.

In general, our findings are in line with other known viral dependencies on Rab1. For example, it is known that the Rab1-activating protein TBC1D20 is involved in the hepatitis C, HIV-1 and HSV replication cycle [47,48,49] and especially Rab1B plays an important role in Ebola virus particle assembly [50] and ER-to-Golgi transport of the vesicular stomatitis virus glycoprotein [28]. The overall less striking influence of Rab1A might be explained by the Golgi-associated localization of GDP-bound Rab1A or by the reduced expression level of the S25N mutant. As opposed to GDP-bound Rab1B, which acts at an earlier stage during the ER–Golgi passage, this would explain the normal maturation and cleavage of S after DN Rab1A-S25N co-expression. Possibly, this makes Rab1A dispensable during early trafficking pathways of SARS-CoV-2 S but more important for peripheral transport processes and particle release (Figure S2). For example, Rab1A is known to be an important host factor for the assembly of the classical swine fever virus [51]. In our experiments, Rab1A-S25N showed a reduced expression level. Consequently, we cannot exclude that this effect might have influenced the results of this study and that an improved expression of this mutant would have led to more striking effects. Thus, further investigations are necessary to improve our understanding of the role of Rab1A in SARS-CoV-2 trafficking. The effect of Rab1 on other SARS-CoV-2 proteins (e.g., M and E) is not yet known.

In sum, our studies indicate that Rab1B is an important factor in SARS-CoV-2 S trafficking and maturation. Elucidation of the exact transport and assembly processes of SARS-CoV-2 might facilitate the development of new vaccines and antivirals.

## Data Availability

All relevant data are within the manuscript.

## Supplementary Materials

The following supporting information can be downloaded at: https://www.mdpi.com/article/10.3390/v15040824/s1, Figure S1: Cleavage of SARS-CoV-2 S can differ among different cell lines, Figure S2: Rab1A and Rab1B are involved in release of SARS-CoV-2 S, Figure S3: Inhibition of rab1B leads to redistribution of SARS-CoV-2 S in infected HEK293^ACE2^ cells, Figure S4: Control Panels of single-expressed S and endogenous GM130 and ERGIC-53 in HuH7 cells.
