# Supplementary material for: Inhibition of Rab1B Impairs Trafficking and Maturation of SARS-CoV-2 Spike Protein
Source: Viruses. 2023 Mar 24;15(4):824. doi: 10.3390/v15040824 (PMC10145535; doi:10.3390/v15040824)
Supplement: Supplementary file 1 [file viruses-15-00824-s001.zip › viruses-2208355-supplementary.pdf]

Supplementary Figure S1

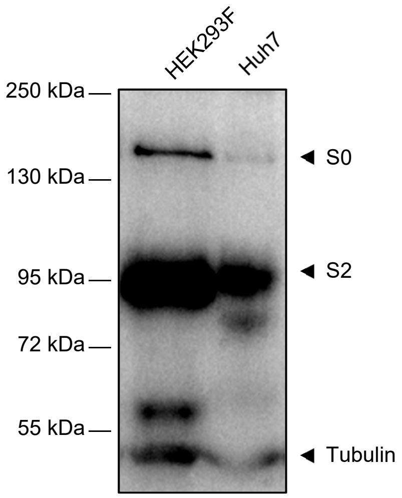

Figure S1. Cleavage of SARS-CoV-2 S can differ among different cell lines. Subconfluent HEK293F and HuH7 cells were transfected with 2 µg C-terminally myc-tagged pCAGGS-S. Immunoblot was stained for S and tubulin.

Supplementary Figure S2

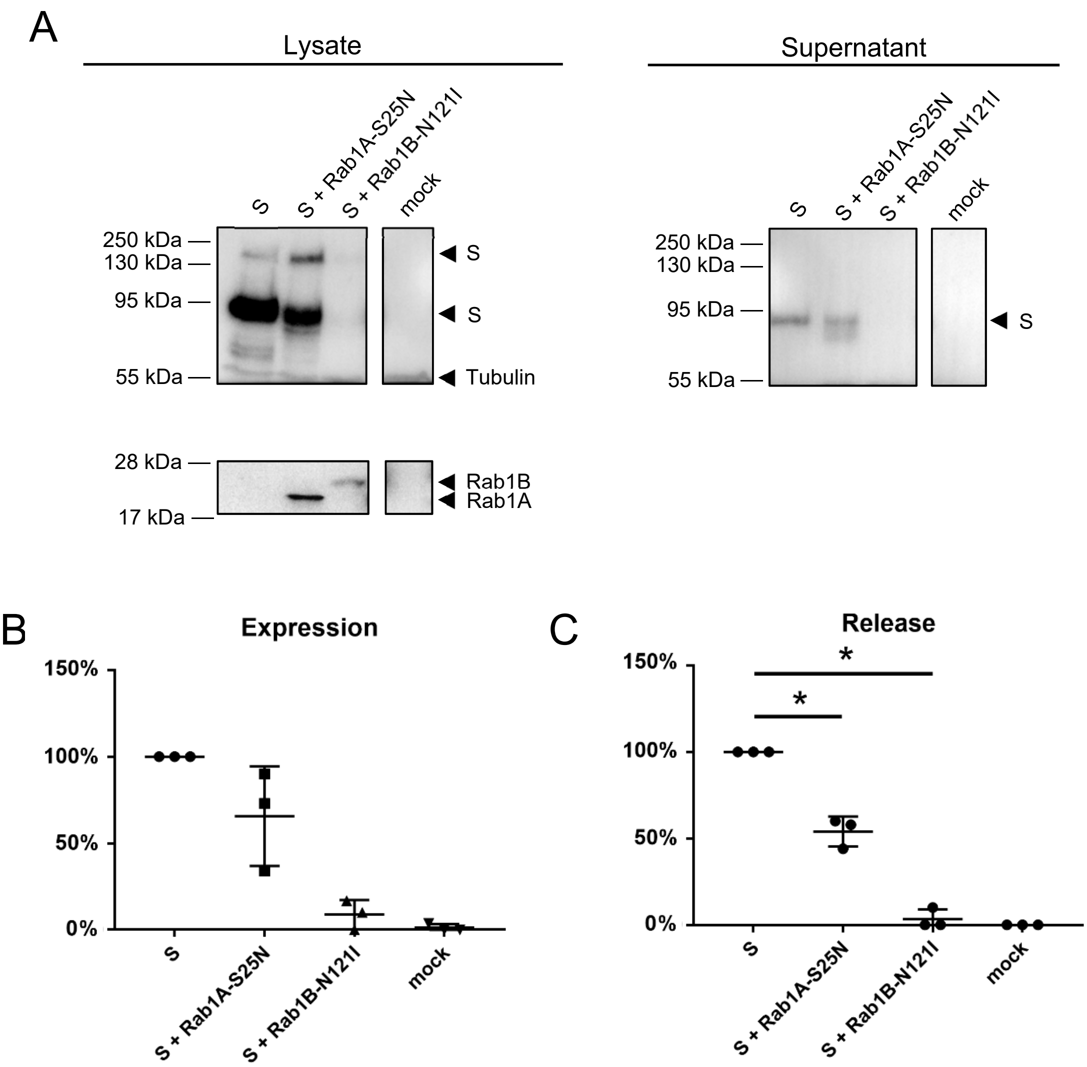

Figure S2. Rab1A and Rab1B are involved in release of SARS-CoV-2 S. (**A**) Subconfluent HuH7 cells were transfected with 1 µg pCAGGS-S and and 3 µg pCMV-Rab1A-myc, pCMV-Rab1B-myc, the corresponding DN mutants or empty vector pCAGGS as a control, respectively. Cell lysates and supernatant were harvested 24 hpt. Released S protein was isolated by ultracentrifugation and resuspended in SDS sample buffer. Immunoblots were stained for S protein, Rab1A/Rab1B (anti-myc) and tubulin. (**B**) Levels of S in the lysate were quantified and normalized to tubulin. (**C**) Levels of released S was quantified and normalized to S in the lysates. The values in the graphs represent triplicates from three independent experiments. Statistical analysis was performed using Mann-Whitney test in R-Studio v.1.3.1073. *: $p < 0.05$

Supplementary Figure S3

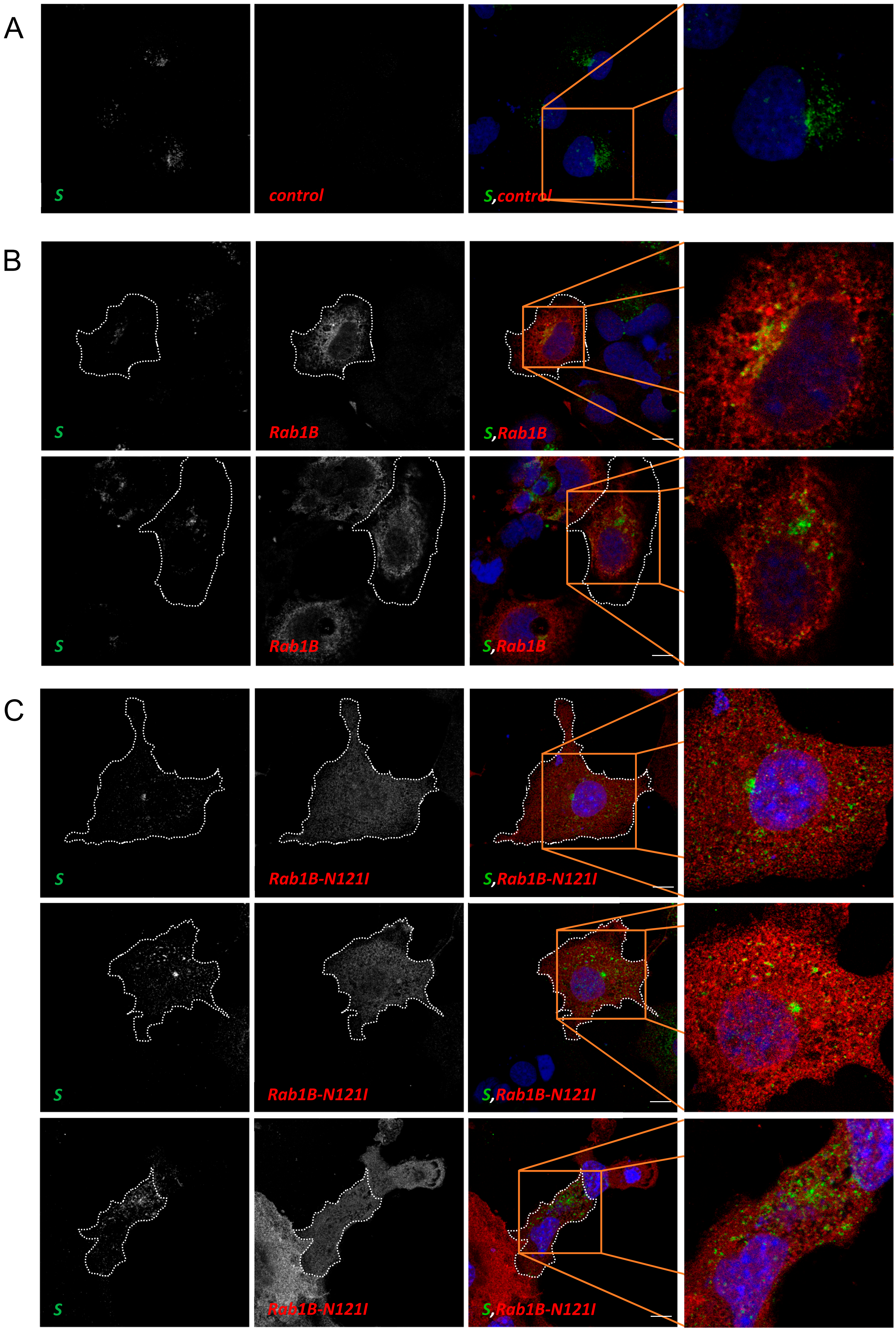

Figure S3. Inhibition of Rab1B leads to redistribution of SARS-CoV-2 S in infected HEK293^{ACE2} cells. Subconfluent HEK293^{ACE2} cells were infected with SARS-CoV-2. Then, cells were transfected with 1000 ng (**A**) empty vector pCAGGS (control), (**B**) pCMV-Rab1B-myc or (**C**) pCMV-Rab1B-N121I-myc, respectively. Next, 24 hpt cells were fixed and stained for SARS-CoV-2 S (green), Rab1B (anti-myc, red) and nuclei (DAPI). Magnified region shows the difference in distribution of S. Scale bars: 10 μm. Data representative for a duplicates of three independent experiments.

Supplementary Figure S4

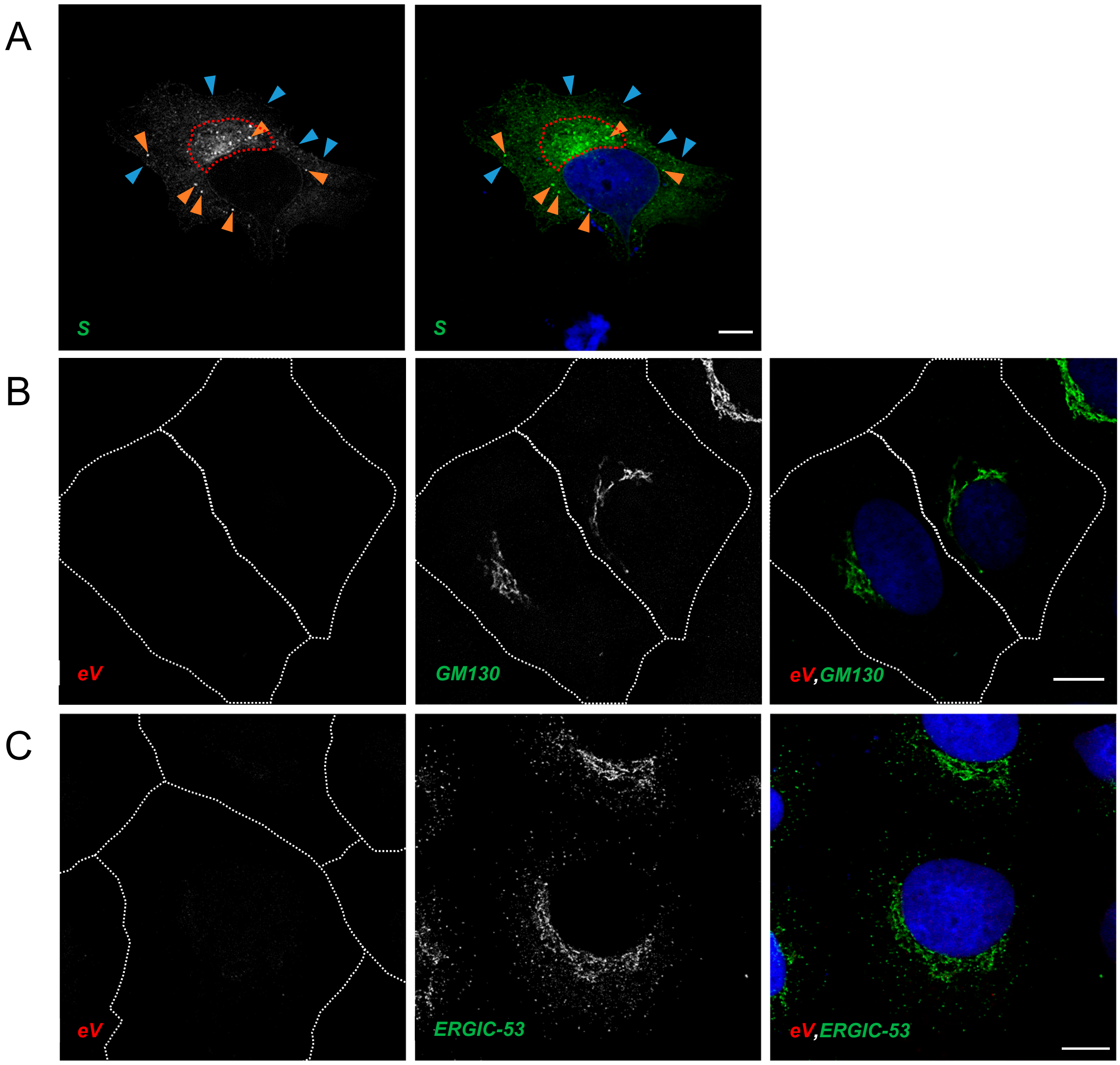

Figure S4. Control Panels of single-expressed S and endogenous GM130 and ERGIC-53 in HuH7 cells. Subconfluent HuH7 cells were transfected with (**A**) 500 ng pCAGGS-S or (**B, C**) 750 ng empty vector (eV) pCAGGS. Next, 24 hpt cells were fixed and stained for nuclei (DAPI), (**A**) SARS-CoV-2 S (green), and (**B, C**) Rab1B (anti-myc, red) as well as (**B**) endogenous GM130, or (**C**) endogenous ERGIC-53, respectively. (**A**) i) Encircled are marks perinuclear accumulation, ii) orange arrows demonstrate vesicle-like structures and iii) blue arrows mark localization at the plasma membrane. Scale bars: 10 µm. Data representative of three independent experiments.
